# Multivariate statistical assessment of a polluted river under nitrification inhibition in the tropics

**DOI:** 10.1007/s11356-017-8989-2

**Published:** 2017-04-13

**Authors:** Thi Thu Huyen Le, Stephanie Zeunert, Malte Lorenz, Günter Meon

**Affiliations:** Department of Hydrology, Water Resources Management and Water Protection, Leichtweiss Institute for Hydraulic Research and Water Resources, University of Braunschweig, Beethovenstr. 51a, D-38106 Braunschweig, Germany

**Keywords:** Vietnam, Dong Nai river, Water quality, Tapioca wastewater, Nitrification, Cluster analysis, Principal component analysis, Discriminant analysis

## Abstract

A large complex water quality data set of a polluted river, the Tay Ninh River, was evaluated to identify its water quality problems, to assess spatial variation, to determine the main pollution sources, and to detect relationships between parameters. This river is highly polluted with organic substances, nutrients, and total iron. An important problem of the river is the inhibition of the nitrification. For the evaluation, different statistical techniques including cluster analysis (CA), discriminant analysis (DA), and principal component analysis (PCA) were applied. CA clustered 10 water quality stations into three groups corresponding to extreme, high, and moderate pollution. DA used only seven parameters to differentiate the defined clusters. The PCA resulted in four principal components. The first PC is related to conductivity, NH_4_-N, PO_4_-P, and TP and determines nutrient pollution. The second PC represents the organic pollution. The iron pollution is illustrated in the third PC having strong positive loadings for TSS and total Fe. The fourth PC explains the dependence of DO on the nitrate production. The nitrification inhibition was further investigated by PCA. The results showed a clear negative correlation between DO and NH_4_-N and a positive correlation between DO and NO_3_-N. The influence of pH on the NH_4_-N oxidation could not be detected by PCA because of the very low nitrification rate due to the constantly low pH of the river and because of the effect of wastewater discharge with very high NH_4_-N concentrations. The results are deepening the understanding of the governing water quality processes and hence to manage the river basins sustainably.

## Introduction

The pollution of surface water in developing and emerging countries is becoming more and more serious in recent years due to rapid industrialization, urbanization, and growth of population. This leads to considerable environmental and social problems such as water quality degradation and risks to public health. One of the countries currently facing this problem is Vietnam, where surface water is frequently used for the purpose of water supply and irrigation (Meon et al. 2014). In order to manage surface water effectively, monitoring programs and a model system are needed to identify water pollution, to detect infringements of regulations, and to enable local authorities to make reliable decisions in the management of water quantity and quality. A model system consists of interacting models for the water balance, the transport of pollutants into receiving water bodies, and the water quality of water bodies (Le et al. 2012; Lorenz et al. 2014). Within monitoring programs, the surface water quality is usually measured over a long time. This results in a huge and unclear data matrix comprised of a large number of physical–chemical parameters, which are often difficult to evaluate and interpret due to their complexity. To solve this problem, different multivariate statistical techniques such as cluster analysis (CA), discriminant analysis (DA), and principal component analysis (PCA) can be applied. These methods have been widely used to reduce the data amount, to interpret and understand water quality data, and to identify possible factors/sources that influence water systems, hence offering a valuable tool for a reliable management of water resources (Krishna et al. 2009; Mavukkandy et al. 2014; Shrestha and Kazama 2007; Singh et al. 2004; Wang et al. 2012).

CA groups objects (monitoring sites) into classes (clusters) on the basis of similarities within a class and dissimilarities between different classes (Singh et al. 2005). It helps to classify the river monitoring sites and to develop future spatial sampling and management strategies. DA can be used in combination with CA to find variables which significantly discriminate between the defined clusters. PCA is a very powerful technique applied to reduce the dimensionality of a data set consisting of a large number of interrelated variables (water quality parameters), while retaining as much as possible the variability present in data set (Singh et al. 2004). With the help of PCA, the correlation between variables can be detected and pollution sources (natural and anthropogenic) can be determined.

Most of the authors used statistical techniques to analyze the temporal and spatial variations of water quality parameters without any detailed examination of particular processes. Among them, Phung et al. (2015) and Wunderlin et al. (2001) used CA, PCA, and DA to evaluate temporal and spatial variations in surface water quality of the Mekong Delta area and the Suquia River basin (Argentina), respectively, dividing the data into wet and dry season for the temporal analysis. Similarly, Singh et al. (2004, 2005) used 24 water quality parameters monitored from 1994 to 2001 to investigate the Gomti River in India by means of these three methods and grouped the data into winter, summer, and monsoon. In Shrestha and Kazama (2007) and Wang et al. (2012), the annual four seasons winter, spring, summer, and autumn were selected for the temporal analysis of the Fuji River basin in Japan and the Xiangxi River basin in China, respectively. Some papers have focused on the analysis of heavy metal pollution on surface water using multivariate statistical techniques (Varol and Şen 2012; Varol 2011; Li and Zhang 2010; Krishna et al. 2009; Jan et al. 2010; Ma et al. 2016), whereby only heavy metals such as Pb, Cu, and Cr were evaluated. CA is often used to analyze the spatial variation of water quality, but some authors also used CA to identify the temporal change in water quality (Wang et al. 2013; Mavukkandy et al. 2014; Xu et al. 2012). Wang et al. (2013) investigated the monthly variation of the Shonghua River in China using CA. In total, 15 water quality parameters collected at six monitoring sites from 2005 to 2009 were used. CA grouped 12 months into three separate clusters, characterized as the low flow period (January and February), high flow period (May, June, July, August, September, and October), and typical mean flow period (March, April, November, and December).

From our knowledge, up to now, no studies have dealt with rivers which are affected by disturbed biochemical processes such as inhibited nitrification. In this paper, the Tay Ninh River, a tropical organically and nutrient-polluted river under iron contamination and under nitrification inhibition, is analyzed using the mentioned statistical methods. In such rivers, the oxidation of ammonium and nitrite is disturbed by environmental factors such as low DO, unfavorable pH, or other inhibitors. This study has three main objectives. The first objective is the evaluation of water quality of the Tay Ninh River to analyze the problems it faces. For the second objective, similarities and dissimilarities between monitoring sites of the Tay Ninh River are extracted and water quality variables responsible for spatial variations, the pollution sources, and parameter interaction of underlying processes in water quality are identified using statistical methods. The third objective is to intensively examine the nitrification inhibition of the Tay Ninh River by means of PCA. For these studies, water quality data of the Tay Ninh River collected in a weekly interval monitoring program from 2009 to 2010 (9120 observations) were used.

## Materials and methods

### Study area

**Fig. 1:**
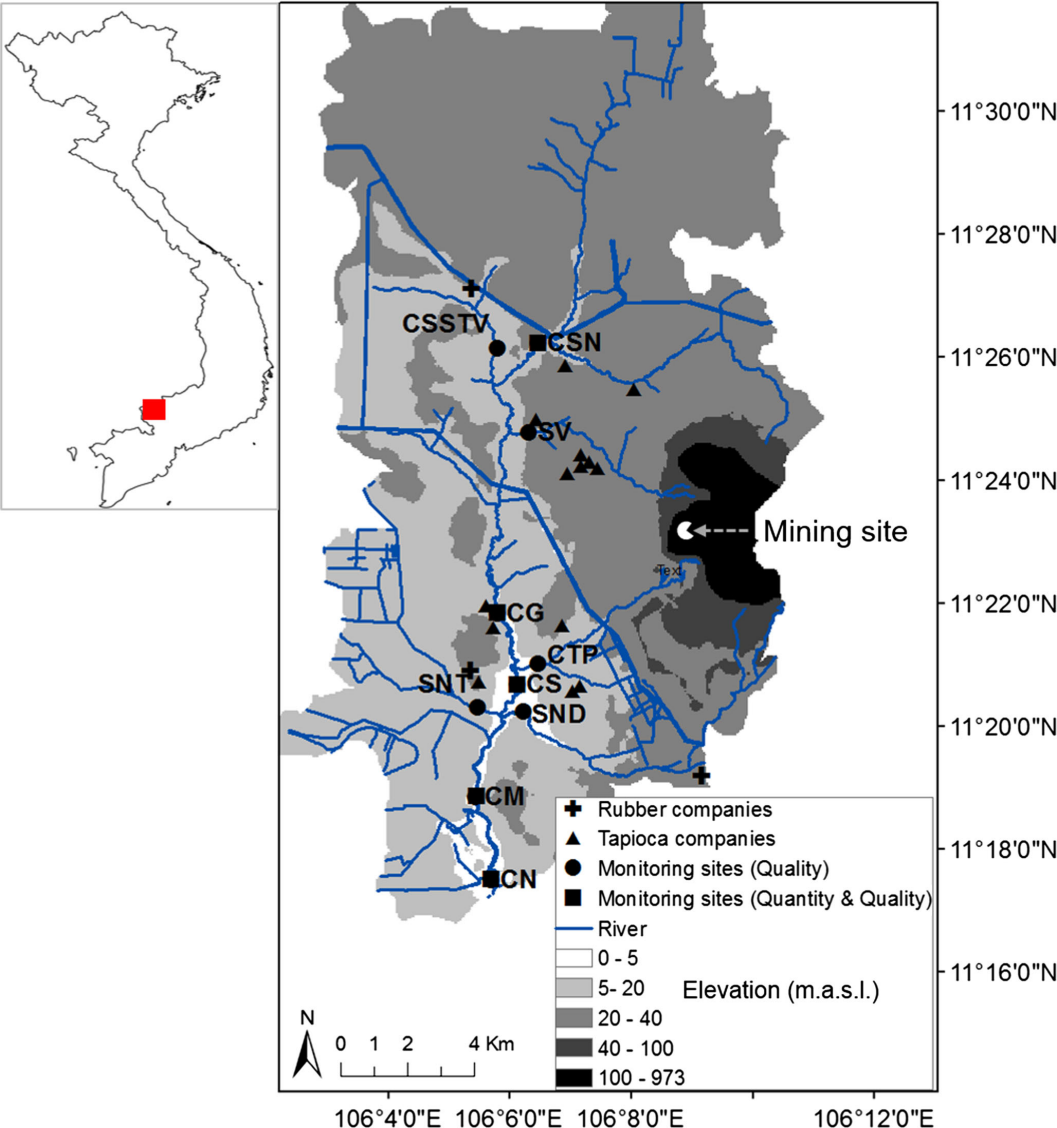
Water quality monitoring sites on the Tay Ninh River. The sites CN, CM, CS, CG, and CSN are at the main river (water quantity and water quality were measured at these sites). The sites CSSTV, SV, CTP, SNT, and SNT are at the tributaries (only water quality was measured)

The Tay Ninh River is a small, highly polluted river of the Dong Nai River basin, one of the largest and most important national river basins in Vietnam. The Tay Ninh River has a catchment of 315 km^2^ and a length of 38 km. The surface width ranges from 10 to 40 m at mean discharge conditions. The terrain of the catchment area is very flat, with the exception of the Nui Ba Den Mountain in the eastern part (Fig. 1). The catchment is affected by a tropical monsoon climate, with a characteristic rainy season from May to November and a dry season from December to April. The mean annual rainfall is 1850 mm, and 80% of which falls in the rainy season. This causes flooding in the rainy season (about 85% of the total water discharge of the river takes place in the rainy season) and droughts and low flows during the dry season (Le et al. 2013). The annual average evaporation is about 1245 mm, and the average air temperature is 27.4 °C. The Tay Ninh catchment is characterized by high agricultural activities. Approximately, 80% of the land within the basin is used for agricultural activities with intense farming of cassava, rubber trees, and rice. Ferralic acrisol is the domimant soil in the catchment with 84%.

The water of the Tay Ninh River is used for the purposes of irrigation and fisheries. Within the catchment, there are numerous industrial companies producing tapioca starch and rubber, which discharge untreated wastewater into the river (Fig. 1). Among these companies, tapioca starch companies are dominant (Fig. 1). For the production of 1 t tapioca starch, about 15 cm^3^ wastewater is produced (Fettig and Pick 2013). In the Tay Ninh catchment, a tapioca company produces 100 t of tapioca starch per day on average. The concentrations of BOD_5_, NH_4_-N, and PO_4_-P of tapioca wastewater have an average concentration of 6700 mg/L, 30 mg N/L, and 58 mg P/L, respectively (Fettig and Pick 2013). This causes a huge amount of highly contaminated organic wastewater in the catchment. This wastewater is also characterized by low pH (around 4.5). The whole wastewater is discharged into a pond system without any technical measures to prevent penetration into the groundwater and is further discharged irregularly into the Tay Ninh River when the storage capacity of the ponds is exceeded (Mai 2006). The uncontrolled release of untreated wastewater, mainly from the tapioca production, causes a severe deterioration of water quality in the Tay Ninh River system. In addition, the rubber production, the intensive agricultural activities (80% of land), and the urban areas (19% of land) also contribute to the water pollution problem. In total, 76,500 inhabitants (data from 2014) live in the catchment, mainly in the areas around stations CN and CM. Domestic wastewater is discharged directly into the river basin without any pretreatment. For all the reasons mentioned above, the river has been highly polluted with ammonium, phosphate, organic substances, and total iron over a long time (Le 2014).

### Monitored parameters and analytical methods

In the Tay Ninh River basin, there are no monitoring stations for measuring continuously water quality and water quantity. Within the research project TAPIOKA financed by the German Ministry of Education and Research (BMBF), 10 monitoring sites, namely Cau Suoi Nuc (CSN), Cau Gio (CG), Cau Sat (CS), Cau Moi (CM), Cau Noi (CN), Cau Sat Suoi Tra Vong (CS STV), Suoi Vang (SV), Cau Tra Phi (CTP), Suoi Nuoc Trong (SNT), and Suoi Nuoc Duc (SND) were installed in the Tay Ninh River basin. These sites were monitored weekly during 15 months from 2009 to 2010. CSN, CG, CS, CM, and CN are located on the main river, and CS STV, SV, CTP, SNT, and SND are on the tributaries (Fig. 1). Because the financing budget for monitoring in the project was not enough for intensive monitoring, the number of sites, the number of monitored parameters, and the monitoring period had to be limited. Therefore, only the water quality (no quantity) of the monitoring sites on the tributaries of the river was measured, and only nine water quality parameters were chosen for analysis in the laboratory. Water samples were collected at a depth of 0.2 m in the middle of the stations using the bridges at the stations as sampling platforms. Water physical parameters such as temperature, pH, electric conductivity, and dissolved oxygen were directly measured in the field using a multiparameter sonde (V2-4 6600, YSI). In situ measurement, sampling, preservation, and transportation were performed carefully. Water sample analysis took place at the Institute of Environment and Resources, Vietnam National University of Ho Chi Minh City. The laboratory of this institute also participates in regular national monitoring programs on environmental control. In total, nine parameters were measured in the laboratory, including BOD_5_, COD_Cr_, TSS, total Kjeldahl nitrogen (TKN), NH_4_-N, NO_3_-N, total phosphorus (TP), PO_4_-P, and total Fe. The analysis of the water samples was performed carefully within 24 h after the sampling with duplicate determination for each water sample. Table 1 lists the analytical methods applied for each parameter.

**Table 1:** Analytical methods applied for each parameter

Parameter	Description	Unit	Method
T	Temperature	°C	V2-4 6600, YSI
pH	pH	–	V2-4 6600, YSI
Cond.	Electric conductivity	μS/cm	V2-4 6600, YSI
DO	Dissolved oxygen	mg/L	V2-4 6600, YSI
BOD_5_	Biochemical oxygen demand	mg/L	5210 (B) APHA 2005
COD_Cr_	Chemical oxygen demand	mg/L	5220 (C) APHA 2005
TSS	Total suspended solids	mg/L	2540 (D)-Solids APHA 2005
TKN	Total Kjeldahl nitrogen	mg N/L	4500-N (C) APHA 2005
NH_4_-N	Ammonium	mg N/L	4500-NH3 (F) APHA 2005
NO_3_-N	Nitrate	mg N/L	TCVN 6180:1996 (ISO 7890-3:1988)
TP	Total phosphorus	mg P/L	4500-P (D) APHA 2005
PO_4_-P	Ortho phosphate	mg P/L	4500-P (D) APHA 2005
Total Fe	Total iron content	mg Fe/L	3500-Fe (B) APHA 2005

### Multivariate statistical methods

Within the framework of the monitoring in the project, organic nitrogen was not measured directly. Instead of organic nitrogen, TKN as the sum of organic nitrogen and ammonium was quantified. The difference between TKN and ammonium results in the concentration of organic nitrogen. Due to high ammonium concentrations and in order to evaluate the organic pollution of the river, organic nitrogen (Norg), instead of TKN, was considered in the statistical assessment. For the multivariate statistical analysis of the catchment’s water quality, two data matrices were used. The first matrix included the parameters pH, conductivity, DO, BOD_5_, COD_Cr_, TSS, organic nitrogen (Norg), NH_4_-N, NO_3_-N, TP, PO_4_-P, and total Fe. For the second matrix to evaluate the nitrification inhibition, pH, DO, NH_4_-N, and NO_3_-N were considered. The water temperature was not included in the analysis due to its spatial and temporal consistency in the catchment which results from the catchment’s location in the tropics. The first data matrix, containing 12 water quality parameters, was subject to three multivariate techniques: CA, PCA, and DA. The second data matrix, for the evaluation of the nitrification inhibition, was analyzed by PCA. DA was applied to raw data, whereas PCA and CA were applied to standardized data (*z*-scale transformation) to avoid misclassifications arising from different parameter units (Singh et al. 2004; Singh et al. 2005; Shrestha and Kazama 2007). All mathematical and statistical computations were made using MATLAB 8.1 (R2013a) and SPSS 23.0.

#### Cluster analysis

CA classifies objects (monitoring sites), so that each object is similar to the others in the cluster with respect to a predetermined selection criterion. The resulting clusters of objects should then exhibit high internal (within cluster) similarities and high external (between cluster) dissimilarities (Shrestha and Kazama 2007). The hierarchical algorithm of cluster analysis is the most common approach. Hierarchical methods are divided into agglomerative and divisive methods. Agglomerative hierarchical methods form clusters sequentially, by starting with the most similar pair of objects (monitoring sites) and forming higher clusters step by step (Wunderlin et al. 2001). The similarity between two objects can be presented by the squared Euclidean distance, which is calculated by the difference between standardized analytical values from both objects. Hierarchical agglomerative analysis includes several methods, which differ in their calculation of proximity between the clusters. Among them, Ward’s method is the most common method in the statistical analysis of water quality (Singh et al. 2005; Shrestha and Kazama 2007). In contrast to the other methods, Ward’s method does not use cluster distances as the factor determining joining clusters. Instead, the total error sum of squares within cluster is calculated to decide the next two clusters merged at each step of the algorithm. In this study, the hierarchical agglomerative clustering using Ward’s method is performed, whereby the similarity between the two objects is calculated using the squared Euclidean distance. The results of the CA are reported as linkage distance *D*_link_/*D*_max_, which represents the quotient between the linkage distance for a particular case divided by the maximal linkage distance; this quotient is then multiplied by 100 as a way to standardize the linkage distance represented on the *x*-axis (Wunderlin et al. 2001; Phung et al. 2015).

#### Discriminant analysis

To perform DA, a prior classification of groups (clusters) of the objects (monitoring sites) is required. This can be done with the help of CA. DA is used to determine variables (water quality parameters) which significantly contribute to the separation of the groups. With the help of DA, the reduction of parameters will be possible. DA technique builds up a discriminant function (DF) for each group using standard, forward stepwise, and backward stepwise modes (Phung et al. 2015; Wunderlin et al. 2001). This function is a linear combination of variables and is calculated using the function below:

$$ f\left({G}_i\right)={k}_i+\sum_{j= i}^n{w}_{i j}{p}_{i j} $$

where *i* is the number of groups (*G*), *k*_*i*_ is the constant inherent to each group, *n* is the number of parameters used to classify a set of data into a given group, *w*_*j*_ is the weight coefficient, assigned by DA to a given selected parameter (*p*_*j*_) (Shrestha and Kazama 2007; Singh et al. 2005). DA delivers a classification matrix to assess the performance of DA. This matrix is simply a table in which the rows are the clusters assigned by DA and the columns are the clusters defined by CA. When prediction is perfect, all measurements will lie on the diagonal. The percentage in this table is the percentage of correct classifications (Shrestha and Kazama 2007). In this case study of DA, the groups previously classified by CA are given into DA. Standard and forward stepwise modes are used to construct discriminant functions for clusters.

#### Principal component analysis

PCA is used to identify patterns in data, their similarities and differences by reducing the number of dimensions and complexity in the data matrix of the independent variables. In PCA, a data set containing correlated variables will be transformed to a new data set containing new orthogonal, uncorrelated variables called principal components (Olsen et al. 2012). In the field of water quality, PCA can be used to detect the correlation between water quality parameters and to determine pollution sources (point and nonpoint pollution). By dividing the data set into different periods, the PCA can also be used to investigate the temporal variations of the water quality and find out the most important pollution sources for each period.

The PCA technique starts with extracting the eigenvalues (EVs) and eigenvectors of the correlation matrix (covariance matrix) of the standardized independent variables. An eigenvalue gives a measure of the significance of principal components. The eigenvectors multiplied by the square root of the eigenvalues produce a matrix of principal component loadings (PCs), which represent the importance of each original variable to a particular component. Therefore, the loadings often provide insight into the relationship of a given PC with a given variable (Olsen et al. 2012). For each component, the number of original variables is equal to the number of principal component loadings. Principal components with the highest eigenvalues are the most significant, and eigenvalues of 1.0 or greater are considered significant (Love et al. 2004; Wang et al. 2013). In this study, only components with eigenvalues higher than 1.0 are retained for evaluation. Principal component scores are calculated by multiplying the PCs with the standardized variables (Olsen et al. 2012). Algebraically, for *p* original variables (water quality parameters) *x*_1_, *x*_2_, …*x*_p_ and *j* sample number (equal score number)

$$ {z}_{i j}={x}_{1 j}{a}_{i1}+{x}_{2 j}{a}_{i2}+\dots +{x}_{pj}{a}_{i p} $$

where *a* is the loading for each variable of extracted components, *z* the component score, and *i* the number of extracted components.

The scores are linear functions of the original variables such that the sum of their variances is equal to that of the original variables (Olsen et al. 2012). After extracting the most important components (EV > 1), the PCA solution is rotated using varimax rotation in order to reduce the contribution of variables with minor significance (Wunderlin et al. 2001).

## Results and discussion

### Evaluation of water quality

After Olsen et al. (2012), when statistical methods are used to evaluate water quality impacts based on chemical and biological data from watersheds, the results will depend upon many factors, including quality of data, treatment, and understanding of data before statistical analysis and interpretation of results. Most of the papers using statistical methods to evaluate the water quality did not adequately address all of these issues (Olsen et al. 2012). The authors of this paper agree with the conclusion of Olsen et al. (2012). Understanding the data first by using the knowledge of the authors without any complex statistical methods is essential and determines the quality of the result interpretation of statistical analysis. Therefore, the water quality data of the Tay Ninh River are first evaluated in this paper using our expertise. After that, statistical methods are applied to further evaluate the data.

**Fig. 2:**
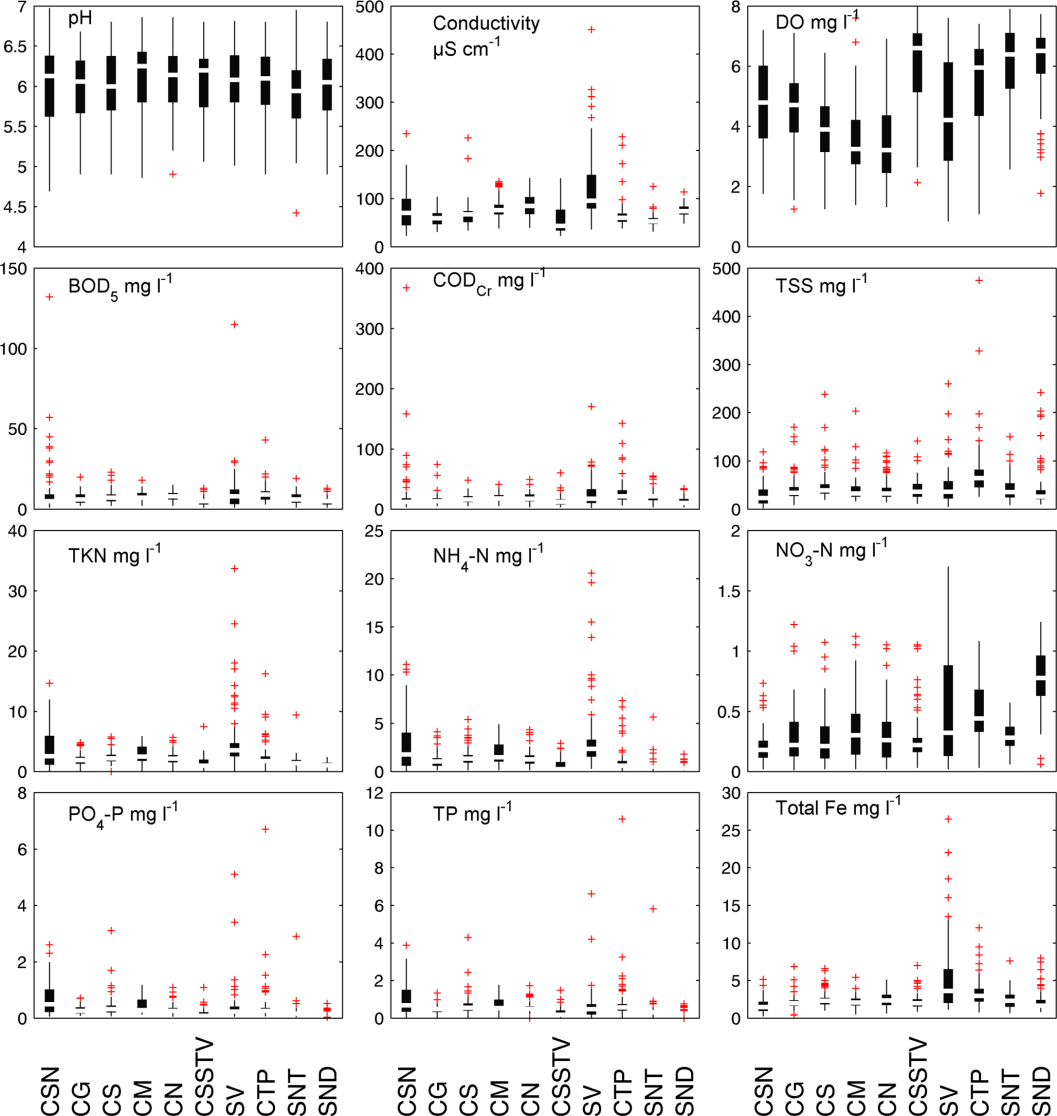
Spatial variation of 12 water quality parameters measured from 2009 to 2010 along the Tay Ninh River and its catchment visualized as *box* and *whisker plots*

The basic statistics of the water quality data set from the monitoring (2009–2010) are summarized in Table 2. Figure 2 shows the spatial variation of the water quality parameters by box and whisker plots. The soils in the catchment are dominated by ferralic acrisols (84%). Ferralic acrisols are strongly weathered acid soils with low base saturation (FAO 2015). The intensive weathering leads to the dominance of highly resistant minerals, such as (hydr)oxides of Fe, Al, and Mn. The clay fraction of ferralic acriols is dominated by low activity clay (LAC) minerals; hence, cations like Ca^2+^, Mg^2+^, and K^+^ exist only in low levels (Do et al. 2006). This causes low pH and high concentrations of total iron in the river. From 2009 to 2010, the measured concentrations of total iron varied from 0.2 to 27 mg/L, whereby the mean values ranged between 1.6 and 5.1 mg/L (Table 2). Among all sites, the sites SV and CTP have the highest concentrations of total iron. The elevated concentrations of total iron at the sites SV and CTP can be related to mining activities at the Nui Ba Den Mountain (Fig. 1) and erosion, while total iron at the other stations is determined by diffuse sources like erosion.

**Table 2:** Range, mean, and standard deviation (SD) of water quality parameters at 10 monitoring sites of the Tay Ninh River basin during 2009–2010

	Sites	CSN	CG	CS	CM	CN	CSSTV	SV	CTP	SNT	SND
pH	Range	4.7–6.7	4.9–6.7	4.9–6.8	4.9–6.9	4.9–6.9	5.1–6.8	5–6.8	4.9–6.8	4.4–6.8	4.9–6.8
	Mean	6.0	6.0	6.0	6.1	6.1	6.0	6.0	6.0	5.9	6.0
	SD	0.5	0.4	0.4	0.4	0.4	0.4	0.4	0.4	0.5	0.4
Cond. (μS/cm)	Range	23–234	31–104	34–225	38–135	40–124	23–141	36–451	39–228	32–125	48–114
	Mean	80.4	59.1	67.4	80.0	85.2	54.3	123.3	67.8	55.3	74.8
	SD	44.0	14.9	28.4	24.8	24.1	26.2	74.1	32.4	12.9	11.4
DO (mg/L)	Range	1.8–7.2	1.3–7.1	1.3–6.4	1.4–7.6	1.3–6.9	2.1–8.0	0.9–7.6	1.1–7.4	2.6–7.9	1.8–7.7
	Mean	4.7	4.6	3.9	3.5	3.4	6.0	4.5	5.4	6.0	6.1
	SD	1.4	1.3	1.2	1.3	1.3	1.3	1.8	1.6	1.3	1.3
BOD_5_ (mg/L)	Range	1–132	2–20	2–23	2–18	2–15	1–13	1–115	3–43	1–19	1–13
	Mean	10.6	6.8	7.8	7.8	7.9	4.9	10.7	9.5	6.8	5.2
	SD	17.5	3.2	4.0	3.3	3.1	2.6	14.0	5.3	3.4	2.7
COD_Cr_ (mg/L)	Range	3–367	5–74	5–48	5–41	3–49	3–60	2–170	6–142	3–55	3–34
	Mean	23.8	15.2	16.8	18.1	18.8	13.1	25.6	27.4	21.3	12.8
	SD	46.2	9.9	7.3	6.7	8.3	8.4	25.7	21.8	10.9	7.6
TSS (mg/L)	Range	2–119	8–170	19–238	16–203	14–117	11–141	5–260	26–474	8–150	10–242
	Mean	30.0	43.0	48.8	41.1	41.4	42.0	47.4	77.8	42.8	44.5
	SD	24.2	28.3	33.6	27.3	25.9	23.2	41.3	63.9	27.4	46.9
TKN (mg N/L)	Range	0.1–14.6	0.5–4.8	0.9–5.7	0.7–5.9	0.2–5.7	0.2–7.5	0.7–34	0.9–16.2	0.5–9.4	0.4–2.4
	Mean	3.9	2.0	2.4	2.9	2.3	1.4	5.1	2.5	1.5	1.1
	SD	3.4	0.9	1.0	1.4	1.0	1.1	5.3	2.3	1.1	0.5
NH_4_-N (mg/L)	Range	0.04–11	0.1–4.1	0.1–5.4	0.1–4.9	0.1–4.4	0–2.9	0.3–21	0–7.4	0.1–5.6	0.1–1.8
	Mean	2.7	1.1	1.5	1.9	1.3	0.6	3.6	1.1	0.6	0.5
	SD	2.8	0.8	1.0	1.2	0.8	0.6	4.0	1.4	0.7	0.3
NO_3_-N (mg/L)	Range	0–0.7	0–1.2	0–1.1	0–1.1	0–1.1	0–1.1	0–1.7	0–1.1	0.1–0.6	0.1–1.2
	Mean	0.2	0.3	0.3	0.3	0.3	0.3	0.5	0.5	0.3	0.8
	SD	0.1	0.2	0.2	0.2	0.2	0.2	0.4	0.3	0.1	0.2
PO_4_-P (mg/L)	Range	0.05–2.6	0.1–0.7	0.1–3.1	0.1–1.2	0.1–1.1	0–1.1	0.1–5.1	0.1–6.7	0–2.9	0–0.5
	Mean	0.7	0.3	0.4	0.4	0.3	0.2	0.4	0.4	0.2	0.2
	SD	0.6	0.2	0.4	0.3	0.2	0.2	0.7	0.8	0.3	0.1
TP (mg/L)	Range	0.05–3.9	0.2–1.3	0.2–4.3	0.2–1.8	0.2–1.7	0.1–1.5	0.1–6.6	0.2–10.6	0.1–5.8	0.1–0.8
	Mean	1.0	0.5	0.7	0.7	0.6	0.3	0.7	0.8	0.4	0.3
	SD	0.85	0.2	0.6	0.4	0.3	0.3	0.9	1.2	0.7	0.1
Fe (mg/L)	Range	0.24–5.2	0.4–6.9	1–6.6	0.5–5.5	0.7–5.1	0.9–7	1.2–27	0.8–12	0.7–7.6	0.8–8
	Mean	1.6	2.1	2.5	2.2	2.5	2.2	5.1	3.4	2.5	2.2
	SD	1.0	0.9	1.1	0.8	0.9	1.0	4.8	1.9	1.2	1.4

**Fig. 3:**
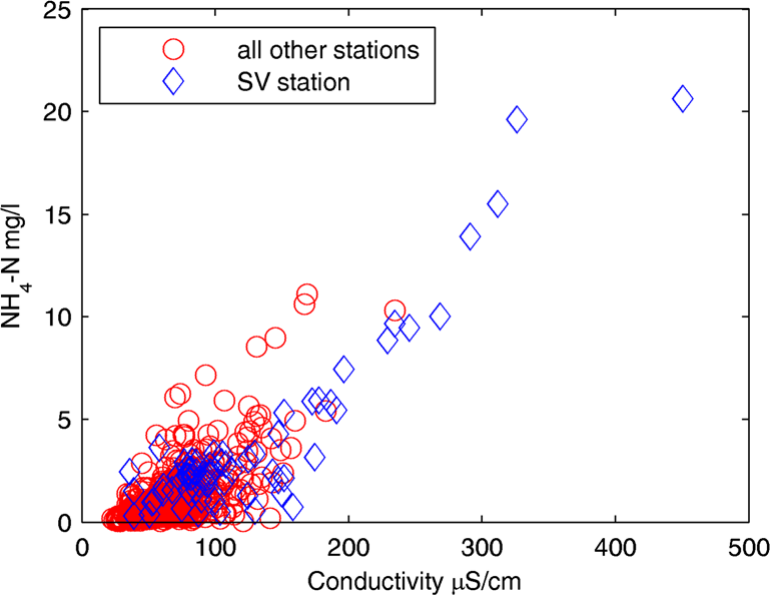
Scatter plots of conductivity vs. ammonium (NH_4_-N) comparing Suoi Vang (SV) station with all other monitoring stations

The river is also contaminated with organic substances and nutrients (Table 2). This causes low dissolved oxygen contents in the Tay Ninh River (Table 2) because aerobic microorganisms deplete the oxygen in the water to extract energy from the organic substances. The two sites SV and CSN are the most polluted sites concerning the pollution of organics and nutrients (Table 2). High BOD_5_ (up to 132 mg/L), ammonium (up to 21 mg N/L), and phosphate (up to 5.1 mg P/L) values are noted at the sites CSN and SV, which are suffering mainly from wastewater discharge of the tapioca production (Fig. 1). The average BOD_5_/COD_Cr_ ratio of the river water is about 0.4 which indicates the biodegradable wastewater influence. A strong correlation between conductivity and NH_4_-N is obvious in Fig. 3. It can be seen that measurements at SV site showed the highest concentrations. The water pollution of the river can be related to industrial wastewater discharge, the domestic wastewater, and the intensive agricultural activities in the catchment.

In Table 2, the range, mean, and standard deviation of pH, DO, NH_4_-N, and NO_3_-N for all sites are shown, whereby many outliers of NH_4_-N were detected (Fig. 2). The maximum NH_4_-N concentrations for the sites SV, CSN, and CTP were 21, 11, and 7.4 mg N/L, respectively. At the sites CG, CS, CM, and CN on the main river, NH_4_-N concentrations up to 5.4 mg N/L were detected. In contrast to the high NH_4_-N concentrations, the NO_3_-N concentrations for all sites are very low with an average between 0.2 and 0.8 mg/L. At the site SV, where the mean NH_4_-N concentration was 3.6 mg N/L, an average NO_3_-N concentration of 0.5 mg N/L was measured. Compared to the high ammonium concentrations with an average concentration over all stations of about 1.5 mg N/L, the nitrate concentrations are rather low with an average of about 0.4 mg N/L. Table 3 shows the ratio of NO_3_-N to NH_4_-N for all sites in the Tay Ninh River, calculated by using the mean concentrations of NO_3_-N and NH_4_-N. It can be clearly seen that the mean nitrate concentrations of all sites are several times lower than the mean ammonium concentrations, except for the site SND. After Wetzel (2001), the ratio of NO_3_-N to NH_4_-N in unpolluted waters, where the nitrification works normally, can have NO_3_-N/NH_4_-N ratios of 25:1. In the Tay Ninh River, these ratios range between 1:2 and 1:12.8, except at the site SND. This indicates an inhibition of the nitrification in the Tay Ninh River basin. Among all stations, the station SND is the only station which shows higher NO_3_-N concentrations although the NH_4_-N concentrations are lower than at the other stations. The mean NO_3_-N concentration at SND is 0.8 mg N/L, and the mean NH_4_-N concentration is 0.5 mg N/L (Table 2). The ratio of NO_3_-N to NH_4_-N shows a better working nitrification at this station (Table 3). It can be assumed that here, the higher DO concentrations and lower NH_4_-N concentrations lead to better conditions for the oxidation from NH_4_-N to NO_3_-N in contrast to the other monitoring sites.

**Table 3:** Ratio of NO_3_-N to NH_4_-N for all sites in the Tay Ninh River, calculated by using the mean concentrations of NO_3_-N and NH_4_-N

Sites	NO_3_-N/NH_4_-N
CSN	1:12.8
CG	1:3.9
CS	1:5.5
CM	1:5.6
CN	1:4.5
CSSTV	1:2.0
SV	1:7.3
CTP	1:2.2
SNT	1:2
SND	1:0.6

Nitrification is a two-step process of the biological oxidation carried out by two different chemolithotrophic bacteria: the ammonium oxidizers (AOBs) and the nitrite oxidizers (NOBs). These bacteria are obligate autotrophic and use energy from nitrification to fix CO_2_. In the first step, ammonium is converted to nitrite. The nitrite is then oxidized to nitrate by nitrite oxidizers (Wrage et al. 2001; Hagopian and Riley 1998; Prosser 1990). The activity of the nitrifying bacteria is affected by certain parameters such as pH, dissolved oxygen, water temperature, salinity, and substrate concentration (Chen et al. 2006; Rheinheimer et al. 1988). Among these parameters, the pH has a strong effect on the nitrification because it not only affects the bacterial growth rates but also modifies the acid base equilibriums NO_2_/HNO_2_ and NH_4_^+^/NH_3_ and delivers substrates for AOB and NOB (Jiménez et al. 2011). This effect was studied by many authors such as De Boer and Kowalchuk (2001), Bae et al. (2002), Grunditz and Dalhammar (2001), and van Hulle et al. (2007). The results of these authors show the optimum pH for AOB and NOB in neutral and slightly alkaline conditions. According to Grunditz and Dalhammar (2001), the optimal pH for AOB is 8.1 and for NOB 7.9. In the studies of Bae et al. (2002) and Jiménez et al. (2011), the optimal pH for AOB as well as NOB is about 8.6. The measured pH of the Tay Ninh River is often under 6.0, and the mean was found between 5.9 and 6.1 (Table 2). These pH values were significantly lower than the given optimal pH for the growth of nitrifying bacteria or nitrification and indicate an inhibition of the ammonium oxidation in the Tay Ninh River due to the unfavorable pH.

Although many researchers reported that lower DO inhibits the growth of AOB and NOB, the critical values of DO recorded in the literatures were different (Peng and Zhu 2006). Bansal (1976) reported that nitrifying bacteria cannot grow if DO in stream waters falls below 0.5 mg/L. Wheaton et al. (1994) suggested the minimum oxygen level in aquaculture nitrification biofilters as 2 mg/L. The mean DO concentrations of the Tay Ninh River range from 3.4 to 6.1 mg/L, whereby concentrations under 2.0 mg/L were found frequently (Table 1). This indicates a moderate influence of DO on the nitrification of the river. Grunditz and Dalhammar (2001) and Bae et al. (2002) proposed that nitrifying bacteria grow optimally in the temperature range between 30 and 40 °C. Since the measured water temperature of the river was between 27 and 32 °C, the effect of water temperature on the nitrification was very low and could be negligible.

To evaluate the nitrification inhibition of the Tay Ninh River, ammonium, nitrite, and nitrate were measured spectrometrically (NOVA 60, Merck) for a short time by the authors in 2013. The nitrite concentrations were rather low with an average of about 0.04 mg N/L. It can be concluded that the nitrification in the Tay Ninh River is inhibited strongly due to low pH and moderately due to low DO. The pH of the river is low over many years. This can cause a negative effect on the growth of the nitrifying bacteria. Based on the evaluation of the water quality data collected during the monitoring, it could not be determined which of the two species, AOB and NOB, is more strongly affected by pH. The reason for this is that the overlaid effect of factors affected the activity of AOB and NOB. In the Tay Ninh River, the ammonium oxidation is dependent on pH and DO. The substrate NH_4_-N for the activity of AOB is mostly adequate due to the high concentrations of ammonium in the river. In contract to AOB, the activity of NOB depends additionally on the availability of substrate NO_2_-N due to the low concentrations of nitrite in the river. In order to determine the sole effect of pH on AOB and NOB separately, a laboratory experiment should be carried out under different pH values. In addition, the approach should be performed under optimum DO, optimum substrate, not only for AOB but also for NOB, and at optimum water temperature.

### Evaluation of the water quality of the Tay Ninh River by means of statistical methods

The first approach was the use of CA, DA, and PCA on the water quality data of the Tay Ninh River basin collected from 2009 to 2010. The aim is firstly to understand and to evaluate the overall water quality of the Tay Ninh River basin. The data set included 12 parameters pH, conductivity, DO, BOD_5_, COD_Cr_, TSS, TKN, NH_4_-N, NO_3_-N, TP, PO_4_-P, and total Fe. CA used a standardized matrix, which was created from the mean values of the evaluated water quality parameter for each site. Defined clusters resulting from CA were used to analyze the spatial similarity of the monitoring sites. DA was applied to the water quality set without any standardization to find variables responsible for classifying the clusters. PCA was applied directly on the standardized matrix including all measured data for all stations to determine the correlation between the parameters and the calculated particular loadings, and thus, the pollution sources could be identified. After the first approach, a second approach was performed to evaluate the nitrification of the Tay Ninh River. For this approach, a second reduced data matrix including only parameters affecting the nitrification such as pH, DO, NH_4_-N, and NO_3_-N was generated. The aim is to evaluate the impact of pH and DO on the ammonium oxidation of the Tay Ninh River.

#### Spatial similarity and site grouping

Cluster analysis classifies the monitoring sites into different clusters. The sites within the same cluster have similar characteristics. It can be assumed that these sites suffer under the same pollution sources such as wastewater discharge or diffuse sources. As pointed out before, two significant pollution sources, affecting the Tay Ninh catchment, are known. The first pollution source is the wastewater discharge of numerous sources in the catchment, and the second one is the diffuse pollution with iron. Due to these two different significant influences, wastewater discharge and iron discharge from the catchment, two cluster analyses were performed. In the first cluster analysis, all 12 parameters were selected. In the second analysis, only 11 parameters were evaluated. Total iron was taken out to evaluate the classification of monitoring sites based on the sole effect of organic biodegradable wastewater components.

**Fig. 4:**
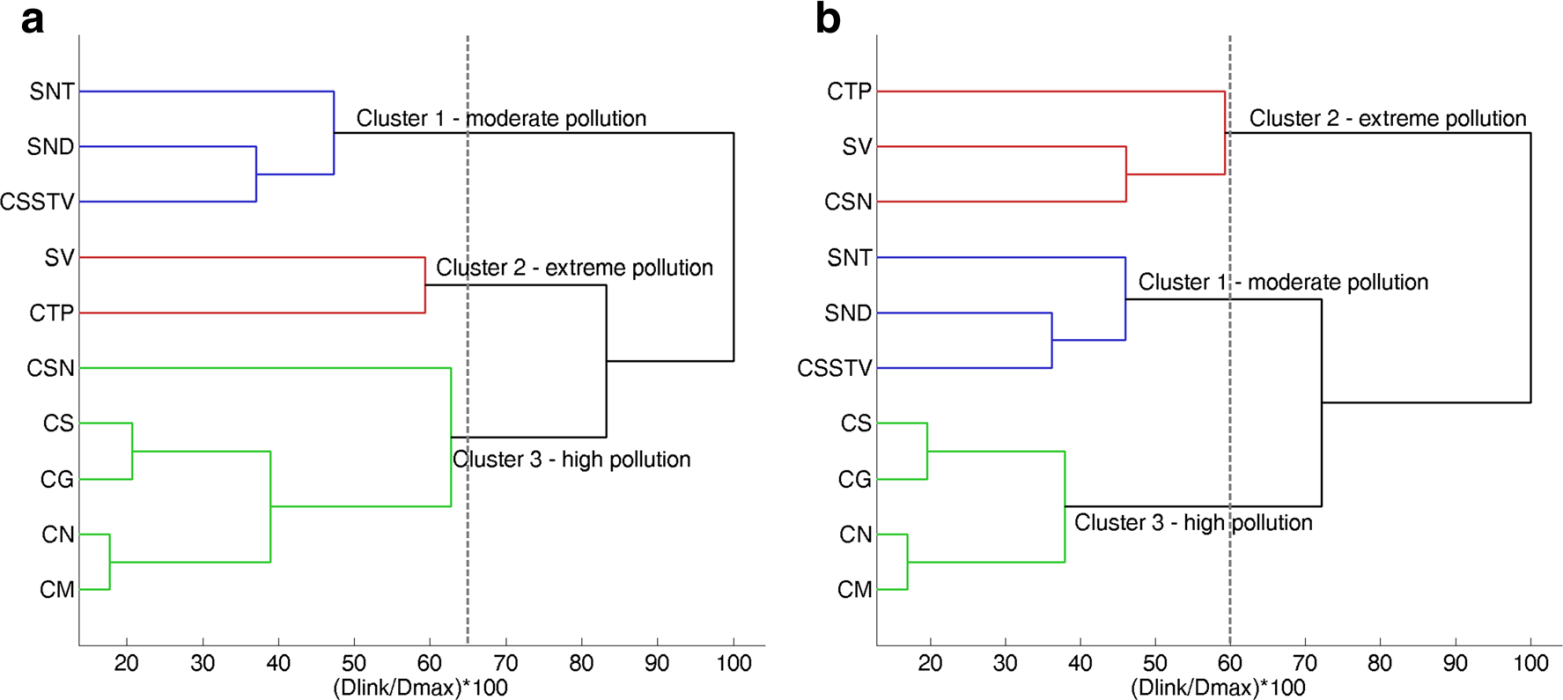
Dendrogram showing clustering of monitoring sites of the Tay Ninh River basin for **a** with total Fe and **b** without total Fe

As results, two dendrograms were generated (Fig. 4). Both of them grouped the 10 monitoring sites into three clusters at (*D*_link_/*D*_max_) × 100 < 65 and at (*D*_link_/*D*_max_) × 100 < 60, respectively. The first dendrogram (Fig. 4a) showed the effect of total iron on the clustering. Cluster 1 (sites SNT, SND, CSSTV), cluster 2 (SV, CTP), and cluster 3 (CSN, CS, CG, CM, CN) correspond to moderate pollution, extreme pollution, and high pollution, especially with regard to the pollution of iron. Organic pollution played an important role in producing the second dengrogram (Fig. 4b). Cluster 1 (STN, SND, CSSTV), cluster 2 (sites SV, CSN, and CTP), and cluster 3 (CS, CG, CM, CN) correspond to moderate pollution, extreme pollution, and high pollution. The difference between the two dendrograms is the grouping of the monitoring site CSN. In the first dendrogram, SV and CTP are heavily contaminated by total iron. Maximum concentrations of total iron up to 27 and 12 mg/L were observed at SV and CTP, respectively (Table 2 and Fig. 2). They were grouped together in one cluster (cluster 2). The site CSN is not much polluted by total iron and was clustered in cluster 3 together with the sites CG, CS, CM, and CN (Fig. 4a). In the second dendrogram (Fig. 4b), where effect of total Fe was removed, SV, CSN, and CTP are the most polluted sites mainly influenced by tapioca wastewater (Fig. 1) and were grouped together in one cluster (cluster 2). The NH_4_-N, PO_4_-P, and BOD_5_ concentrations of these sites were extremely high and differed from the other sites. NH_4_-N, PO_4_-P, and BOD_5_ concentrations at SV were measured up to 21 mg N/L, 5.1 mg P/L, and 115 mg/L (Table 2 and Fig. 2), respectively. At the site CSN, NH_4_-N and BOD_5_ reached concentrations up to 11 mg N/L and 132 mg/L, respectively. The site CTP is less polluted in comparison with SV and CSN. The maximum concentrations of NH_4_-N, PO_4_-P, and BOD_5_ at this site were 7.4 mg N/L, 6.7 mg P/L, and 43 mg/L, respectively.

Cluster 1 in the first dendrogram is almost identical to cluster 1 in the second dendrogram. The two clusters consist of three monitoring sites (SNT, SND, CSSTV). In the sub-catchments of these sites, there are several rubber companies discharging wastewater from the production into the tributaries (Fig. 1). Although the amount of this wastewater is much less than the amount of wastewater from the tapioca production, this wastewater is also polluted with NH_4_-N and organic substances. Furthermore, these sites are located in agricultural-dominated areas with rice and annual crop fields. Therefore, they are moderately contaminated with nutrients and organic substances due to wastewater discharge from the rubber production and from nonpoint discharge over many years. As mentioned above, cluster 3 in the first dendrogram differs from cluster 3 in the second dendrogram in classifying the site CSN. Both of the clusters contain the monitoring sites CN, CM, CS, and CG, whereby their division into two separate sub-clusters is clearly evident. They are located on the main river, two in the middle (CS, CG), and two downstream (CN, CM). The downstream sites CN and CM were grouped in one sub-cluster, while the other sub-cluster includes the sites CG and CS. When including total Fe in the analysis, all sites along the main river were grouped in one cluster.

#### Identification of significant variables discriminating between clusters

The spatial variations in water quality were further evaluated through DA. DA was performed on raw data after dividing the data set into three spatial groups based on the results of CA with and without total Fe (Fig. 4). In total, 12 and 11 parameters were included in the analysis. Two methods were used to obtain the discriminant function coefficients, standard mode, and forward stepwise mode. When using standard mode, all parameters are included. In forward stepwise mode, variables are included step by step beginning with the more significant until no significant changes are obtained (Shrestha and Kazama 2007). For this study, at each step, the variable that minimized the overall Wilks lambda was entered into the analysis. DFs and classification matrices (CMs) obtained for the standard and stepwise mode are shown in Tables 4, 5, 6 and 7. For the DA including total Fe, the standard and stepwise DA mode constructed DFs, including 12 and 7 parameters, respectively (Table 4). Forward stepwise mode indicates that conductivity, DO, NH_4_-N, NO_3_-N, PO_4_-P, TP, and total Fe are the most significant discriminating parameters in space. The classification matrices assign 74.2 and 74.5% of cases correctly for standard and stepwise mode, respectively (Table 5).

**Table 4:** Classification function coefficients for discriminant analysis of spatial variations in water quality of the Tay Ninh River (with total Fe)

Parameter	Standard mode			Stepwise mode		
	Group 1	Group 2	Group 3	Group 1	Group 2	Group 3
pH	36.180	36.401	35.681			
Conductivity	0.163	0.149	0.141	0.133	0.118	0.109
DO	5.619	5.201	4.522	3.640	3.205	2.586
BOD_5_	0.114	0.111	0.196			
COD	−0.013	−0.028	−0.055			
TSS	0.042	0.043	0.045			
Norg	−0.634	−0.329	−0.842			
NH_4_-N	−3.337	−2.592	−2.818	−2.412	−1.633	−1.869
NO_3_-N	1.249	3.424	−0.117	4.182	6.337	2.975
PO_4_-P	−1.817	−10.194	−4.755	3.758	−4.142	1.836
TP	2.595	8.330	5.975	0.719	6.067	3.173
Total Fe	2.051	2.351	1.726	1.003	1.297	0.706
Constant	−134.297	−136.047	−124.281	−18.165	−18.355	−11.433

**Table 5:** Classification matrix for discriminant analysis of spatial variation in water quality of the Tay Ninh River (with total Fe)

Cluster	% Correct	Clusters assigned by DA		
		Cluster 1	Cluster 2	Cluster 3
Standard mode				
Cluster 1	78.2	133	19	18
Cluster 2	55.7	28	64	23
Cluster 3	79.1	37	26	238
Total	74.2	198	109	279
Stepwise mode				
Cluster 1	76.6	134	18	23
Cluster 2	56.0	29	65	22
Cluster 3	80.3	34	26	245
Total	74.5	197	109	290

**Table 6:** Classification function coefficients for discriminant analysis of spatial variations in water quality of the Tay Ninh River (without total Fe)

Parameter	Standard mode			Stepwise mode		
	Group 1	Group 2	Group 3	Group 1	Group 2	Group 3
pH	35.160	35.047	34.910			
Conductivity	0.181	0.162	0.159	0.137	0.117	0.116
DO	5.122	4.596	4.044	3.471	2.948	2.421
BOD_5_	0.142	0.147	0.230			
COD	−0.029	−0.048	−0.070			
TSS	0.078	0.078	0.078			
Norg	−0.576	−0.282	−0.861	0.351	0.480	0.055
NH_4_-N	−2.896	−2.153	−2.458	−2.183	−1.401	−1.726
NO_3_-N	−2.151	−1.115	−2.924	3.309	4.304	2.534
PO_4_-P	−2.188	−8.604	−5.651	2.614	−3.102	0.177
TP	2.666	8.265	5.760	1.875	6.768	4.257
Constant	−127.963	−125.699	−120.019	−16.675	−15.017	−10.444

**Table 7:** Classification matrix for discriminant analysis of spatial variations in water quality of the Tay Ninh River (without total Fe)

Cluster	% Correct	Clusters assigned by DA		
		Cluster 1	Cluster 2	Cluster 3
Standard mode				
Cluster 1	78.2	133	12	25
Cluster 2	41.8	49	71	50
Cluster 3	73.8	34	31	183
Total	65.8	216	114	258
Stepwise mode				
Cluster 1	79.1	140	8	29
Cluster 2	40.4	50	69	52
Cluster 3	73.8	31	35	186
Total	65.8	221	112	267

When removing the parameter total Fe from the DA, the standard DA mode and stepwise DA mode constructed DFs, including 11 and 7 parameters, respectively (Table 6). Forward stepwise mode indicates that conductivity, DO, NH_4_-N, NO_3_-N, Norg, PO_4_-P, and TP are the most significant discriminating parameters in space. The classification matrices assign 65.8% of cases correctly for both modes (Table 7).

In both cases with and without total Fe, only seven parameters are necessary to differentiate 74.5 and 65.8% of the measurements. The results are not improved significantly when using all variables as suggested by standard DA mode (Tables 5 and 7). For both cases, conductivity, DO, NH_4_-N, NO_3_-N, PO_4_-P, and TP were selected. The difference between the two cases is the inclusion of the parameters Norg and total Fe. BOD_5_ and COD were not selected by DA as discriminating parameters in space. Instead of BOD_5_ and COD, Norg and TP were chosen as factors presenting the organic pollution. For DA including total Fe and excluding total Fe, cluster 2 showed for both methods the worst classification results. The water quality at the stations SV, CTP, and CSN (for DA excluding total Fe), which are included in these clusters, shows strong temporal variations resulting from the irregular discharge of wastewater by the surrounding industries and from the mining activities. As mentioned in “Study area” section, wastewater from the tapioca production is discharged into the river when the storage capacity of the ponds is exceeded and it is temporal independent. This is leading to difficulties when classifying single measurements into specific groups.

Factors affecting the nitrification such as DO are important in the discrimination of the monitoring sites. DO concentrations in cluster 1 for DA including total Fe and excluding total Fe are higher, and organic contents and nutrients are lower in contrast to the stations in the other two clusters. Cluster 1 comprises the stations SNT, SND, and CSSTV with lower pollution in comparison to the other stations. In summary, including total Fe into the analysis leads to much better classification results for both methods. This can be explained by the low spatial variations for all monitoring sites except the sites SV and CTP (Fig. 2). The results of DA for the analysis with total Fe for cluster 2, which comprises the sites SV and CTP, is low. Both the standard and the forward stepwise mode DFs for this cluster rendered the lowest CMs assigning 55.7 and 56.0% of the cases correctly, respectively (Table 5).

#### Data structure determination and source identification

The PCA was performed on the normalized data set (12 variables) for 10 monitoring sites. The size of the input data matrix [variables × measurements] was [12 × 760]. To examine the suitability of the data set for PCA, Kaiser–Meyer–Olkin (KMO) and Bartlett’s tests were performed. KMO is a measure of sampling adequacy that indicates the proportion of variance which is common variance, i.e., which might be caused by underlying factors (Parinet et al. 2004). Backhaus et al. (2015) classified the KMO as “marvelous,” “meritorious,” “middling,” “mediocre,” “miserable,” and “unacceptable” corresponding to a KMO of ≥0.9, ≥0.8, ≥0.7, ≥0.6, ≥0.5, and <0.5. In this study, the KMO value is 0.78 and indicates that PCA is possible. Bartlett’s test of sphericity indicates whether the correlation matrix is an identity matrix, which would indicate that variables are unrelated (Shrestha and Kazama 2007). The significance level after Bartlett which is 0 in this study (less than 0.05) indicates that there are significant relationships among variables.

The aim of PCA is to find correlations between the original variables and PCs and to define the pollution sources which affect the water quality of the Tay Ninh River basin. The PCs are constrained between −1 and +1. High negative and positive loadings mean that the variables are important for the defined pollution source and conversely. Liu et al. (2003) classified the component loadings as “strong,” “moderate,” and “weak,” corresponding to absolute loading values of >0.75, 0.75–0.5, and 0.50–0.30, respectively. For the water quality data set used in this study, four PCs were extracted using PCA. In Table 8, the loadings of the principal components as well as eigenvalues, total, and cumulative variance in % are shown. Eigenvalues measure the significance of the PCs; the higher the eigenvalues, the more significant are the loadings. The sum of all eigenvalues equals the sum of the variances of the original variables. Only PCs with an eigenvalue higher than 1.0 are retained for evaluation. Among the four eigenvalues, the first principal component (PC1) has the highest value and is the most important PC. In total, 73.86% of the variables variance can be described by the four PCs.

**Table 8:** Loadings of the variables on the first four principal components after varimax rotation for the data set measured from 2009 to 2010 of the Tay Ninh River

Parameters	PC1	PC2	PC3	PC4
pH	0.27	−0.24	−0.15	−0.12
Conductivity	*0.81*	0.20	0.22	−0.04
DO	−0.23	0.04	−0.24	*0.77*
BOD_5_	0.32	*0.87*	0.13	−0.05
COD	0.17	*0.89*	0.20	−0.05
TSS	−0.08	0.33	*0.75*	0.17
Norg	0.36	*0.73*	0.17	−0.10
NH_4_-N	*0.91*	0.13	0.10	−0.08
NO_3_-N	−0.03	−0.16	0.29	*0.79*
PO_4_-P	*0.85*	0.33	−0.07	−0.14
TP	*0.77*	0.46	0.02	−0.19
Total Fe	0.19	0.11	*0.85*	−0.11
Eigenvalue	3.25	2.66	1.60	1.35
Total variance [%]	27.05	22.16	13.37	11.28
Cumulative variance [%]	27.05	49.21	62.58	73.86

Values greater than 0.5 are marked in italics

The first principal component PC1 shows strong loadings for conductivity, NH_4_-N, PO_4_-P, and TP. The conductivity is a measure for the whole dissolved dissociated substances (electrolytes) in water and thus an indication of the mineral content of the water (Hütter 1992). The main electrolytes (the cations Na^+^, K^+^, Mg^2+^, and Ca^2+^ and anions Cl^−^, NO_3_^−^, HCO_3_^−^, and SO_4_^2−^) and accompanying substances (the cations NH_4_^+^ and Fe^2+^ and anions NO_2_^−^ and HPO_4_^2−^) are electrolytes measured by the conductivity in natural water bodies (Hütter 1992). Due to the dominance of ferralic acrisols in the catchment, as a result of intense weathering, main cations, such as Ca^2+^, Mg^2+^, and K^+^, are washed out and exist only in very low levels (Do et al. 2006; FAO 2015). The main electrolytes are therefore only present in small concentrations in the Tay Ninh River water. In contrast, the concentrations of accompanying substances such as NH_4_^+^ and HPO_4_^2−^ are high (Table 2 and Fig. 2). Both ions are charged, leading to a strong influence on conductivity and a very good correlation between conductivity, NH_4_-N, PO_4_-P, and TP. The PC1 represents the nutrient pollution group and is in agreement with the results of the preliminary investigation illustrated in Fig. 3. The second principal component PC2 explains 22.16% of the total variance and shows strong positive loadings for the variables BOD_5_, COD_Cr_, and moderate loading for Norg. Naturally, these variables are highly correlated due to their overall representation of organic components in the water. Thus, this PC basically represents the organic pollution group.

**Fig. 5:**
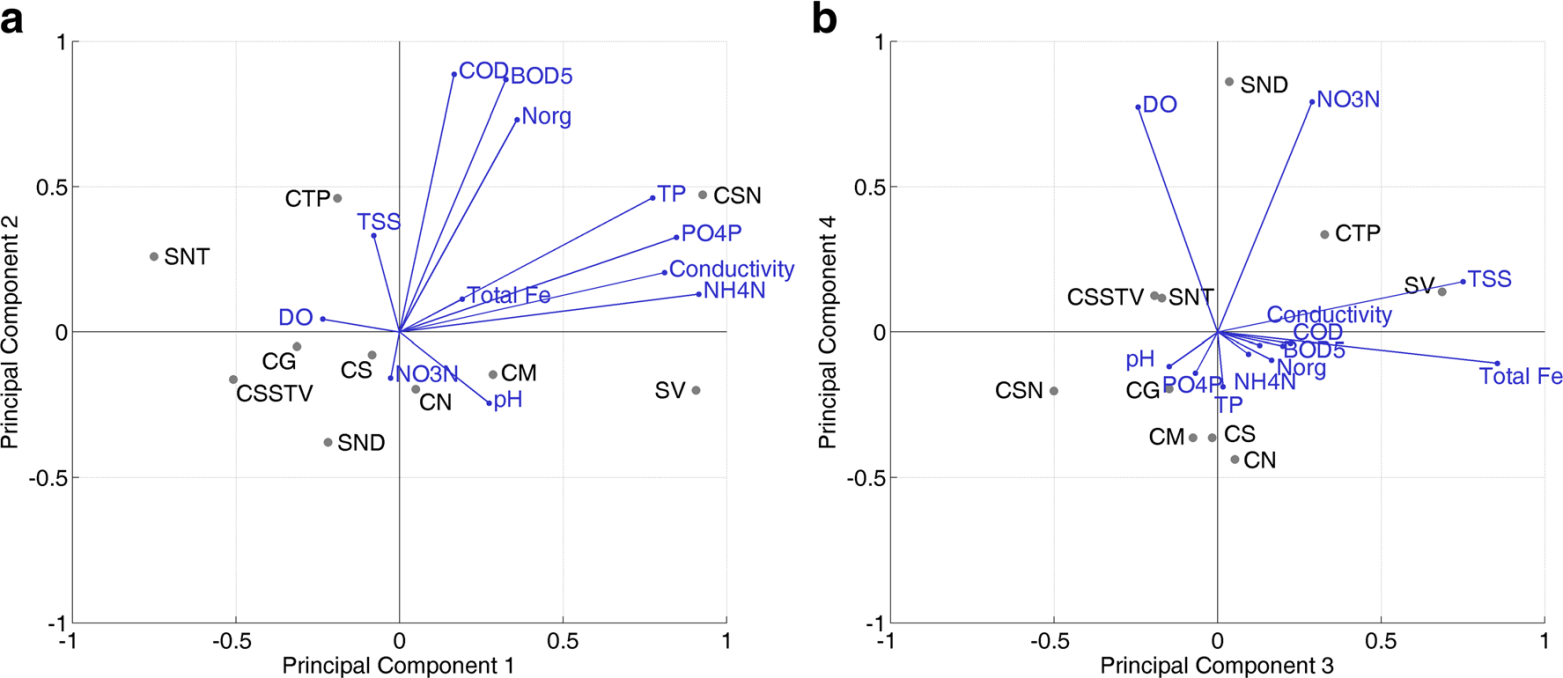
Biplot for **a** PC1 and PC2 and **b** PC3 and PC4 showing the loadings and mean normalized scores of each station based on the data set measured from 2009 to 2010 of the Tay Ninh River

**Fig. 6:**
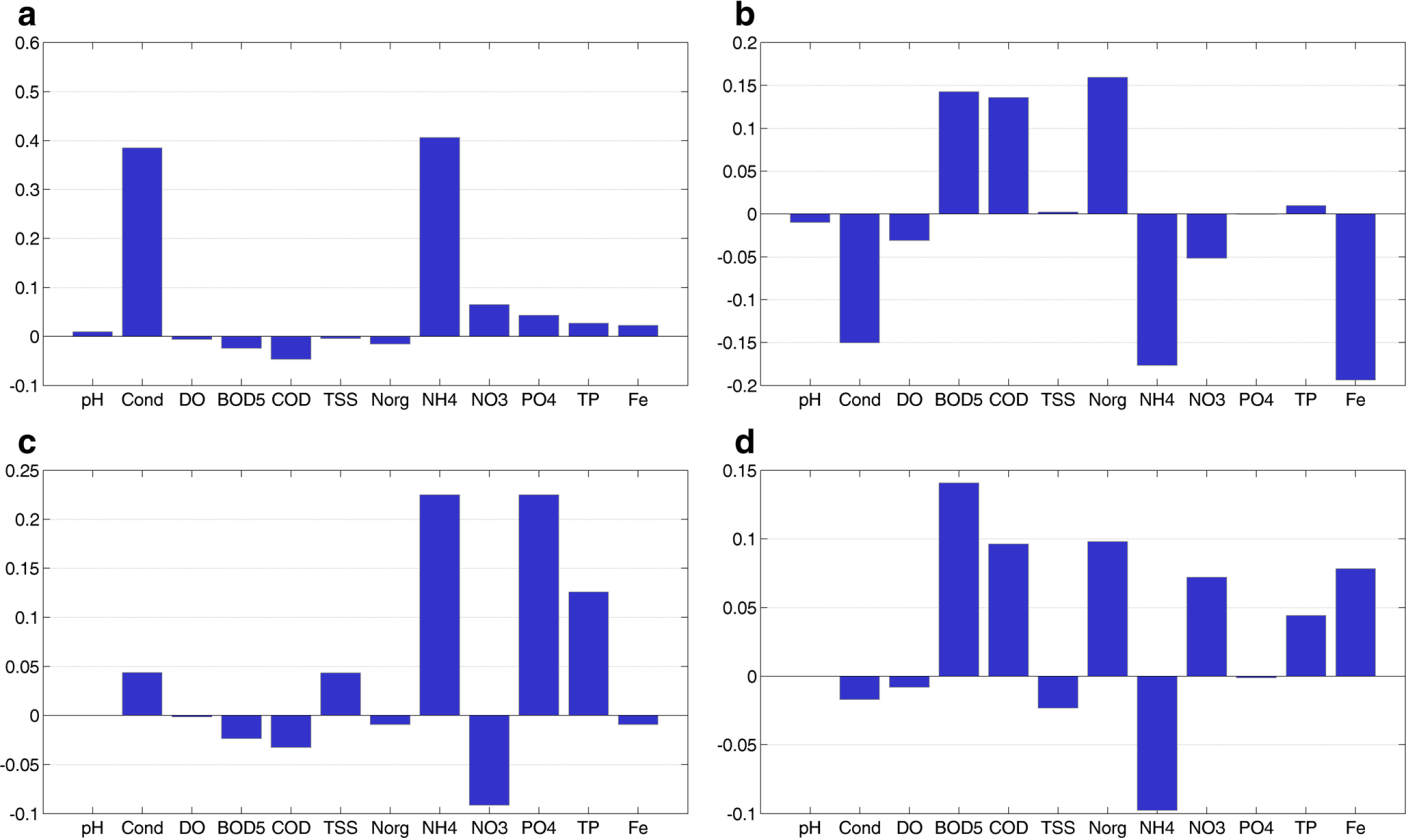
Contribution of variables to the mean principal component scores of PC1 and PC2 for **a**, **b** SV and **c**, **d** CSN

The loadings and mean normalized scores for each station of PC1 and PC2 are presented in a biplot in Fig. 5a. While the station CSN corresponds to both PC1 and PC2, the station SV is closely related only with PC1. Actually, it is expected that the station SV is also related with PC2 like the station CSN because these two stations are heavily contaminated with organic components as well as nutrients as shown in the cluster analysis and evaluated in “Evaluation of water quality” section. To understand the reason for this positioning, the contribution of variables to the mean principal component scores of PC1 and PC2 for SV and CSN was calculated. Figure 6a–d shows the mean sub-scores calculated separately for each variable. According to the equation presented in “Principal component analysis” section, these sub-scores are the separate terms of this equation. The sum of these sub-scores results in the mean principal component score for each station. It can be seen that the parameters conductivity, NH_4_-N, and total iron highly influence the PC2 score in a negative direction (Fig. 6b), although the loadings for these variables are small. This is a result of the very high concentrations of these parameters at the station SV (Table 2). The separated mean sub-scores of PC2 for the station CSN are shown in Fig. 6d. It can be seen that the variables conductivity, NH_4_-N, and total Fe only have a small effect on PC2. In contrast to the station SV, the variables BOD_5_, COD, Norg, NO_3_-N, and TP of the station CSN have the most influence on the calculation of the PC2 score, thus leading to a very positive score on the PC2 axis. Besides the stations SV and CSN, the station CTP is also highly polluted and is positively related with PC2. The results in Fig. 5a also show that the stations SNT and CSSTV are negatively related to PC1 due to the low NH_4_-N concentrations of these sites in comparison to other sites in the catchment. The mean NH_4_-N concentrations of these two sites are identical and equal to 0.6 mg N/L (Table 2), although some outliers were detected due to wastewater discharge from the rubber production and the agricultural activities as mentioned in the cluster analysis. In summary, both principal components PC1 and PC2 represent mainly the influence of wastewater discharge and agricultural activities in the catchment area.

The third principal component PC3 has strong positive loadings for TSS and total Fe. The soils in the catchment are largely characterized by ferralic acrisols. As a result of erosion, soil particles and iron are transported into the river leading to high TSS and total Fe concentrations. The scores of the stations CTP and SV in Fig. 5b are closely related to PC3. This result matches the results of the cluster analysis. The reason for the high concentration of total iron of these two stations is the mining activity on the Nui Ba Den Mountain in the east of the study area (Fig. 1). Wastewater produced from the washing of the raw materials is discharged into the two tributaries, particularly into SV. PC3 represents the soil erosion by surface water runoff and the impact of mining activity within the catchment. Figure 5b also shows that the site CSN is least influenced by PC3 due to its lower concentrations of total Fe. The mean total Fe concentration of this site is 1.6 mg/L and significantly below the mean concentrations of other sites (Table 2).

The fourth principal component PC4 shows strong positive loadings for DO and NO_3_-N. These two variables are closely related concerning the biological processes nitrification and denitrification. Nitrification is dependent on the availability of DO. Low DO will lead to an inhibition of the process. As already mentioned in “Evaluation of water quality” section, the nitrification of the Tay Ninh basin is inhibited. One of the reasons is the moderately low DO concentrations. The station SND is closely related to PC4 showing a higher correlation of DO and NO_3_-N (Fig. 5b). This confirms the nitrification evaluation performed in “Evaluation of water quality” section. An intensive evaluation of the nitrification in the Tay Ninh River is performed in “Evaluation of the nitrification in the Tay Ninh River by means of statistical methods” section of this paper.

Several pollution sources have been extracted for the Tay Ninh River using PCA. The main pollution sources are untreated wastewater discharge, mainly from the tapioca production and the rubber production in the catchment. Total iron is a significant parameter contributing to the pollution as result of soil erosion and mining activity.

### Evaluation of the nitrification in the Tay Ninh River by means of statistical methods

Up to now, no papers have dealt with the statistical analysis of the nitrification to find out which factors have an impact on the nitrification and how these factors influence the ammonium oxidation and nitrite oxidation in the river systems. Most of the papers statistically analyzed nearly all the parameters measured by monitoring programs. Thus, the effect of parameters inhibiting the nitrification will be overlaid by other parameters/processes or pollution sources which might be dominating when analyzing the water quality of the investigated river systems.

Based on the results of the monitoring programs and on the first statistical evaluation, the nitrification of the Tay Ninh River is inhibited strongly by low pH and moderately by low DO. For the statistical assessment of the nitrification, only measured parameters which directly affect the nitrification were chosen for the analysis. These were pH, DO, NH_4_-N, and NO_3_-N. The influence of water temperature was negligible due to its variation around the optimum for nitrifying bacteria and due to its spatial and temporal consistency in the catchment. The PCA was performed on the standardized data set (four variables) for all 10 monitoring sites. The KMO value of this analysis is 0.54, and the Bartlett value is 0.0. Both values indicate that PCA is possible. PCA renders one PC with an eigenvalue higher than 1.0 accounting for 36.88% of total variance (Table 9). This PC has strong positive loadings for DO, negative moderate loadings for NH_4_-N, and positive moderate loadings for NO_3_-N. This shows a clear negative correlation between DO and NH_4_-N and a positive correlation between DO and NO_3_-N representing the natural oxygen dependence of the nitrification.

**Table 9:** Loadings of the experimental variables on the first principal component after varimax rotation for the data set measured from 2009 to 2010 of the Tay Ninh River

Parameters	PC
pH	−0.385
DO	*0.751*
NH_4_-N	*−0.634*
NO_3_-N	*0.601*
Eigenvalue	1.475
Total variance (%)	36.88
Cumulative variance (%)	36.88

Values greater than 0.5 are marked in italics

The nitrification process is subject to the availability of DO. The oxidation of ammonium produces nitrite. Nitrite is oxidized further to nitrate. The theoretical oxygen requirements according to the nitrification stoichiometric equations are 3.43 mg for the oxidation of 1 mg NH_4_-N and 1.14 mg for the oxidation of 1 mg NO_2_-N (Chen et al. 2006). This indicates that oxygen is consumed during the nitrification activities. Thus, the ammonium reduction is negatively and the nitrate production is positively correlated with the availability of DO. Many researchers reported that a lower DO might inhibit the growth of AOB and NOB (Peng and Zhu 2006; Bae et al. 2002; Munz et al. 2011). After Bansal (1976), the nitrifying bacteria cannot grow if the oxygen contents fall below 0.5 mg/L in stream waters. Peng and Zhu (2006) proposed dissolved oxygen half-saturation coefficients of 1.2–1.5 mg/L for AOB and NOB. Half-saturation coefficients are concentrations at which the nitrification rate is half the maximum rate. In the Tay Ninh River, the DO concentrations are relatively low with a mean concentration of 4.8 mg/L (Table 2), whereby concentrations under 2.0 mg/L were found frequently. This indicates a moderate influence of DO on the nitrification in the Tay Ninh River. This influence is detected by means of PCA as shown in Table 9.

The effect of pH on NH_4_-N and NO_3_-N is not clear in this analysis (Table 9). The reason for this is the constantly low pH of the Tay Ninh River. The mean pH of the river is 6.0, whereby a pH between 4.4 and 5.0 was found frequently. A great number of investigations have demonstrated the pH effects on nitrification (Chen et al. 2006). After Grunditz and Dalhammar (2001) and Jiménez et al. (2011), the relative activity at pH 6.0 of ammonium oxidizing bacteria is about 15% and of nitrite oxidizing bacteria is 0%, respectively. However, these authors investigated the nitrification in wastewater treatment plants. The nitrification in river systems was rarely examined up to now. After Wetzel (2001), the nitrification proceeds very slowly in acidic river systems, so that most of the time, only low quantities of nitrate are found in comparison with ammonium. In rain-acidified lakes, pH 5.4 was identified as the lower limit for nitrification (Jeschke et al. 2013; Rudd et al. 1998). It can be concluded that the nitrification in the Tay Ninh River is inhibited in large parts strongly due to the relatively constant low pH, although its influence could not be recognized by means of PCA due to the very low nitrification rate in the Tay Ninh River and due to the excessive effect of untreated wastewater discharge with very high NH_4_-N concentrations.

## Conclusions

In this study, different multivariate statistical techniques were successfully applied to assess spatial variation in water quality, to determine the main sources/factors responsible for variations in water quality, and to identify the factors inhibiting the nitrification in the Tay Ninh River. Monitoring results showed that the river is slightly acidic and suffers under organic wastewater discharge, iron pollution, and nitrification inhibition. High ammonium concentrations and low nitrate concentrations of the river were caused by an inhibition of nitrification strongly due to low pH and moderately low DO over many years.

CA was carried out to analyze spatial variation for two cases: with total iron (12 parameters) and without total iron (11 parameters). For both cases, CA statistically clustered 10 water quality stations of the Tay Ninh River into three groups based on physical properties such as conductivity and chemical properties such as pH, DO, BOD_5_, COD, TSS, organic nitrogen, ammonium, nitrate, TP, phosphate, and total iron. These three groups corresponded to extreme pollution, high pollution, and moderate pollution based on the interacting effect of iron discharge and wastewater discharge (with total iron) and based on the sole effect of wastewater discharge (without total iron). CA indicated that the stations have a strong clustering structure. Stepwise DA used only 7 parameters, instead of 12 parameters, to differentiate between the stations with and without total iron. The assignation for these two cases (with and without total iron) was about 74.5 and 65.8% right, respectively. The important discriminating parameters are conductivity, DO, NH_4_-N, NO_3_-N, PO_4_-P, Norg, TP, and total Fe. Applying PCA to the data set, four main sources/factors responsible for variations in water quality were identified. The first PC was interpreted as nutrient pollution due to its high correlation with conductivity, NH_4_-N, PO_4_-P, and TP. The second PC represented the organic pollution, because it is associated with the changes of BOD_5_, COD, and Norg. These two first PCs reflected the effect of wastewater discharge mainly caused by the tapioca starch production in the catchment. The iron pollution of the river was illustrated in the third PC which had strong positive loadings for TSS and total Fe. The effect of DO on the nitrification was explained in the fourth PC. In this PC, the nitrate production was positively correlated with the concentration of oxygen as the natural condition of the nitrification.

The factors inhibiting the nitrification, such as pH and DO, were further investigated by means of PCA. The results emphasized that DO affected moderately the oxidation of NH_4_-N and the production of NO_3_-N in the Tay Ninh River. An important result is that the influence of pH on the nitrification could not be detected by PCA because of the constantly very low nitrification rate in the Tay Ninh River and because of the impact of wastewater discharge with very high NH_4_-N concentration. This low nitrification rate is mainly due to the constantly low pH.

From the results of CA, it can be seen clearly that CA is useful to evaluate spatial variation of monitoring networks by classifying water quality monitoring sites into different characteristic clusters. In combination with DA, water quality parameters strongly discriminating between the clusters can be defined. This helps to reduce sampling sites and observed parameters in monitoring networks without losing significant information of the river basin. PCA provides a reliable tool to identify sources of pollution and to understand the parameter interaction of underlying processes in water quality. By analyzing the loadings and mean normalized scores for each monitoring station, the correlation between principal components and monitoring stations can be described.

As investigated in this paper, the organic pollution and nutrients were mainly introduced by the tapioca production. Furthermore, the rubber production, the urban areas, the mining activity, the agricultural activities, and erosion play also an important role in the river pollution. As a consequence, controlling of the tapioca wastewater discharge is primarily important. The intensive agricultural and mining activities and intense erosion lead to high concentrations of total iron and nutrients. Therefore, diffuse pollution should be paid more attention in the water quality management of the river. Furthermore, controlling of wastewater discharge from rubber production and the urban areas is also necessary. This paper helps to better understand the nitrification problem of the investigated river. The nitrification activities in the Tay Ninh River should be further investigated to determine the maximum nitrification rate in dependence on different pH.

The results from the multivariate statistical assessment of the water quality help to understand the water quality of investigated river basins and to correctly calibrate a model system for an integrated watershed management. As mentioned at the beginning of “Introduction” section, such a model system is needed to simulate effects of different treatment facilities on point sources and to deal with management practices on diffuse sources from agricultural activities. In doing so, the interactions of the measures and the overall impact on the water quality can be quantified at any location of the river system, and suitable planning variants can be elaborated.
